# Spatial data collection and qualification methods for urban parks in Brazilian capitals: An innovative roadmap

**DOI:** 10.1371/journal.pone.0288515

**Published:** 2023-08-10

**Authors:** Anne Dorothée Slovic, Claudio Kanai, Denise Marques Sales, Solimar Carnavalli Rocha, Amanda Cristina de Souza Andrade, Lucas Soriano Martins, Débora Morais Coelho, Anderson Freitas, Mika Moran, Maria Antonietta Mascolli, Waleska Teixeira Caiaffa, Nelson Gouveia

**Affiliations:** 1 School of Public Health, University of São Paulo, São Paulo, Brazil; 2 Department of Preventive Medicine, University of Sao Paulo Medical School, São Paulo, Brazil; 3 Observatory for Urban Health in Belo Horizonte, School of Medicine, Federal University of Minas Gerais, Belo Horizonte, MG, Brazil; 4 Institute of Collective Health, Federal University of Mato Grosso, Cuiabá, MT, Brazil; 5 Center of Data and Knowledge Integration for Health (CIDACS), Salvador, BA, Brazil; 6 School of Public Health, University of Haifa, Haifa, Israel; King Abdulaziz University, SAUDI ARABIA

## Abstract

Urban parks have been studied for their effects on health and the environment. Accessing park data from reliable and comparable sources remains challenging, reinforcing the importance of standardized search tools, notably in Latin America. We designed a systematized methodology to identify processes of accessing, collecting, verifying, and harmonizing urban park spatial data in all Brazilian capitals included in the Urban Health in Latin America (SALURBAL) project. We developed a research protocol using official and non-official sources combining the results of Google Maps (GMaps) points and OpenStreetMap (OSM) polygons–GMaps-OSM. Descriptive analyses included the frequency of the distribution of parks before and after harmonization stratified by data source. We used the intraclass correlation coefficient (ICC) to assess agreement in the area between official and GMaps-OSM data. Official data were obtained for 16 cities; for the remaining 11 capitals, we used GMaps-OSM. After verification and harmonization, 302 urban parks were obtained from official data and 128 from GMaps-OSM. In a sub-study of the 16 cities with official data (n = 302 parks), we simulated a collection of non-official data using GMaps-OSM and OSM only. From GMaps-OSM, we obtained 142 parks, and from OSM, 230 parks. Statistical analysis showed a better agreement between official data and OSM. After completing verification and harmonization, the complete dataset (official and GMaps-OSM) included 430 urban parks with a total area of 145.14 km^2^. The mean number of parks across cities was 16, with a mean size area of 0.33 km^2^. The median number of parks was nine, with a median area of 0.07 km^2^. This study highlights the importance of creating mechanisms to access, collect, harmonize, and verify urban park data, which is essential for examining the impact of parks on health. It also stresses the importance of providing reliable urban park spatial data for city officials.

## 1. Introduction

The recognition of urban greenspaces as an essential driver of sustainability is increasing among researchers and policymakers, who identify positive associations between green spaces, health and well-being [1, 2]. Greenspaces have different forms, definitions, and purposes. They are usually characterized by a dedicated space, natural or not, having multiple purposes and usages [3]. Within the urban context, a prevailing form of greenspace includes parks, commonly described as a green area that combines vegetation, regardless of size, with built infrastructure such as playgrounds and amenities open to the public. Usually, they are more extensive than squares [4, 5] and differ from other forms of urban greenspaces, such as natural reserves or conservation units, in that they may or may not be open to the public [6].

Research focusing on urban parks investigates the presence, proximity, degree of greenness, and use of urban parks with enhanced health and increases in the practice of physical activities [7]. The health benefits include decreased risk of cardiovascular diseases, prevention of premature mortality, and improvements in well-being, social interaction, and mental health [7–12]. Further, co-benefits of urban parks include mitigating the impacts of urban violence, reducing air and noise pollution, and temperature reduction [13–17].

Research on urban green areas in Latin America is increasing. However, few studies focus specifically on urban parks. Moran et al. [18] reported that higher park usage is associated with proximity and better-surrounding infrastructure in some Latin American (LA) cities. When offering good infrastructure, such as walking paths, urban parks were associated with increased physical activities [9, 19]. Furthermore, urban inequities are reflected in park inaccessibility, uneven spatial distribution, and obstacles to leisure opportunities [20, 21].

Studies on urban parks in Brazil are scarce, partly due to the scarcity of reliable data on size, form, spatial location, vegetation levels, coverage (green, gray, sand), purpose, use, and accessibility, as well as a lack of consensus on what constitutes an urban park. Given its vast and diverse territory, Brazil has relevant potential to provide insightful research on this topic. It also presents the potential of using various sources of information, such as official data and alternative sources that, in turn can create an opportunity for standardized cross-city research on parks. Therefore, this study aims to provide a methodology to obtain spatial urban park data for Brazilian capitals, exploring data collection options and tools to verify the quality of the data and its harmonization.

## 2. Methods

### 2.1 Study area and sample selection

Our study consisted of 27 geographic units, of which 26 were Brazilian state capitals and one was the Brazilian Federal district, all included in the Urban Health in Latin America (SALURBAL) project. The selection was based on the fact that these capitals can offer better urban infrastructure, public administrative arrangements, and, presumably, more accessible data. Brazilian capitals are characterized by predominantly tropical climates, except for the Northeastern and Midwest regions, which have a semi-arid climate [22]. The Northeastern and Northern regions have the highest number of capital cities. At the same time, the Southeast and Southern regions include some of the most populated cities with the highest Gross Domestic Product (GDP). Detailed information is in the S1 Table.

We limited our sample selection to urban parks located within each municipal administrative spatial unit, excluding Conservation Units (CSU) and other categories of greenspaces (forests, cemeteries, agroecological living centers, different sorts of protected areas not covered by the Conservation Units National System (SNUC) and parks not yet implemented). The CSU are green areas characterized by sizable territorial extension to preserve vegetation and biodiversity [23]. Usually, their territorial border is greater than the municipality’s administrative limits. Their governance and management level vary, being shared with the city, state, or federal government or belonging to one instance.

### 2.2 Urban park data collection steps

#### 2.2.1 Spatial data collection of urban parks

**Fig 1:**
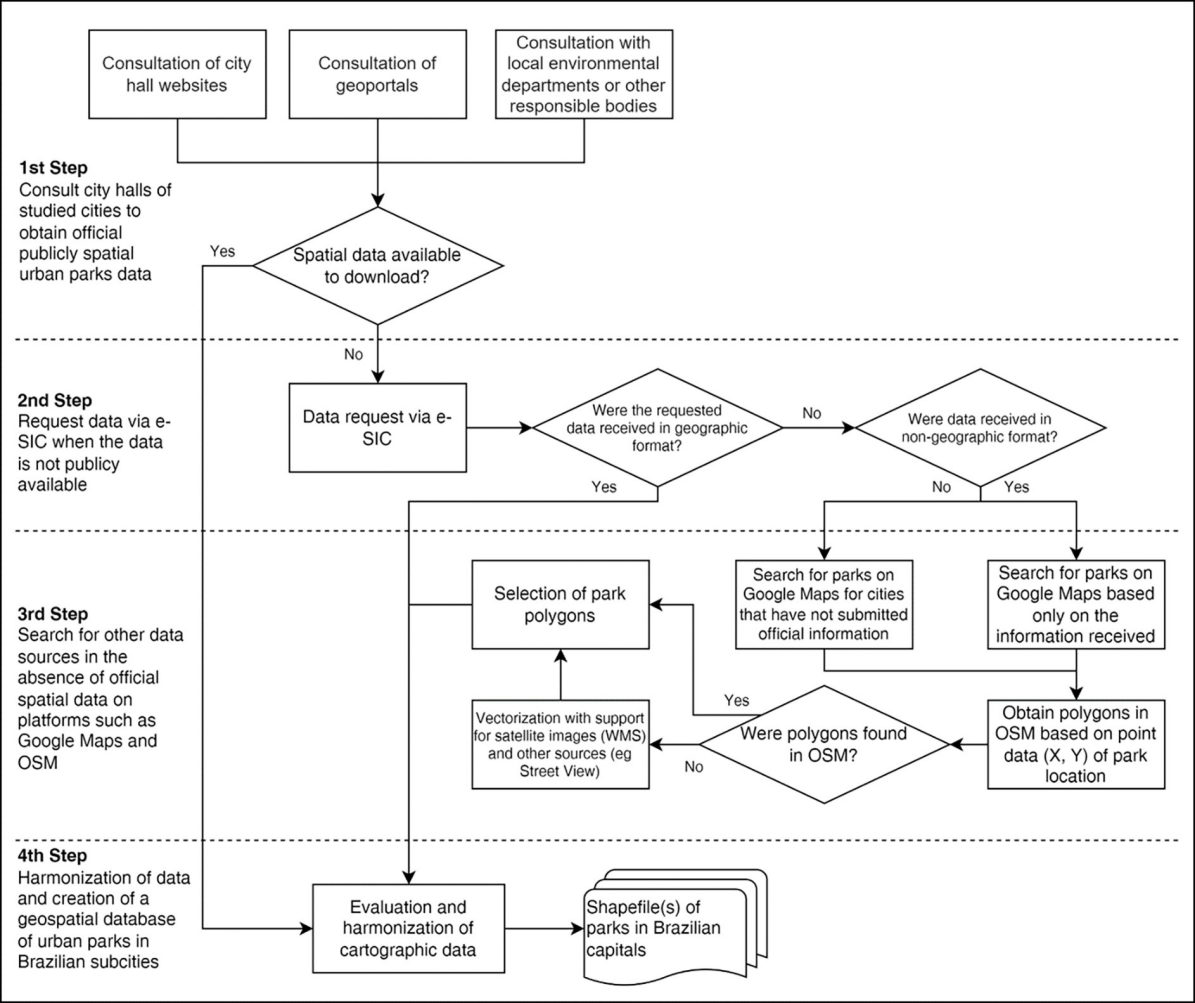
Flowchart of spatial urban park data collection.

We created a systematized methodology for access, verification and harmonizing of the urban park’s spatial data, as illustrated in Fig 1. The first step consisted of consulting and verifying official spatial data for urban parks in vector format available for downloading on websites and geoportals belonging to governmental institutions, city halls, or municipal secretariats related to the environment or urban planning [24].

In the absence of available public data with direct access, a second step was taken by outreaching directly to City Halls using the Brazilian Access to Information Law (Law 12.527, November 18th, 2011) by the Service of Electronic Information System to (e-SIC). e-SIC (hereby Electronic System of Citizens’ Information Service) is an on-line system that centralizes all the requests for information supported by Access to Information Law (Law 12.527/2011), allowing any natural or legal person to request public information, monitor the deadlines and receive answers from public entities where the request is made [25]. A formal letter was written requesting the vector data for urban parks and sent to the responsible bodies in each capital.

In cases where no official response was obtained or did not correspond to vector data, as requested, we proceeded to a third step, where we searched for spatial information on Google Maps (GMaps) and OpenStreetMap (OSM). In cities where we received or found only textual information (such as address, name, coordinates, and description) on parks, we used them to support data collection on GMaps and OSM. Otherwise, we relied on the results provided by GMaps searching for the term "park" followed by the name of the respective city. We checked each park’s result and excluded those not urban parks, although it had the word parks in it, such as shopping malls, residential condominiums, amusement parks, squares, cemeteries, parking lots, and other commercial places. This verification was done via the place’s name and visual inspection of satellite images and using Google Street View. GMaps only helped identify park points as it does not have information on polygons. To do so, we used OSM to capture polygons and define park boundaries using the tag “leisure = park”[26]. The combination between the results of GMaps points and OSM polygons reinforced the existence and location of a park.

In some cases, the polygons found were incomplete, a known issue to the OSM platform. When it was impossible to obtain the data in polygon format, it was necessary to vectorize the park area. We then turned to the manual vectorization strategy to capture the urban park features on the combined textual information found in official sources, such as name, address, neighboring streets, and total area size. This procedure was carried out with the support of visual inspection of satellite images via Google Earth and using Google Street View to verify the physical limits of the park.

Finally, we unified all the polygon data gathered in steps 1 and 3 and proceeded with the harmonization to turn them into a single shapefile of urban parks. The first part of this process consisted of identifying and removing from official sources the Conservation Units polygons, publicly available on the Ministry of Environment website. We compared the urban park shapefiles to the CSU shapefiles, checked for overlaps, and removed them when a match occurred. We also removed topological errors and duplicates. Finally, polygons were harmonized considering cartographic projection, datum, and attribute table composition. A metadata set was made for the final single shapefile.

#### 2.2.2 Comparison of non-official and official spatial data

For the 16 capital cities for which we obtained official spatial data, we compared the number of parks and their area to the non-official data obtained from GMaps and OSM. This process followed two distinct approaches:

The first, named GMaps-OSM, where we replicated the third step of the flowchart (GMaps to obtain the list of parks and OSM to obtain the polygons), simulating the cases where we had no official information on urban parks. The spatial database generated was overlapped with the Conservation Units shapefile and the polygons that matched were removed.

The second approach, named OSM, was done by overlapping the harmonized official data with the polygons extracted from OSM for the same cities to check if there would be any substantial differences compared to the first method.

#### 2.2.3 Statistical analysis

Data accuracy was verified by comparing the frequency of the distribution of parks before and after harmonization. To do so, we calculated the number and total area in km^2^ of parks for each capital and stratified by source (harmonized official, GMaps-OSM, and OSM). For each capital and group by the data source, we calculated the agreement percentage corresponding to the number of concordant parks divided by the total number of harmonized parks. We used the intraclass correlation coefficient (ICC) and its 95% confidence intervals (95%CI) to assess the reliability (Interrater reliability) between the official harmonized source and OSM for each capital. The ICC was estimated by absolute agreement and obtained by the Two-Way Mixed-Effects Model [27] using Stata version 12.0 (StataCorp LP, College Station, USA).

## 3. Results

### 3.1 General description

We managed to obtain official spatial data for 16 cities and non-official spatial data for the remaining 11 cities. Table 1 presents the descriptive analysis before and after harmonization for the 16 cities with official spatial data comparing with results obtained using GMaps and OSM. We observed parks overlapping with CSU in ten cities; therefore, they were removed, corresponding to 21.5% of the official park polygons we had access to. In addition, other types of green spaces were also removed from official data in some cities (17.2%). As a result, the number of parks was reduced by 38.5%, and the park area by 69.8%. We identified 302 urban parks in the 16 capitals analyzed from the harmonized official data. The Southeast region had the highest number of parks and the greatest availability of official spatial data while the most extensive park area was found in Brasilia.

**Table 1:** Number and area of urban parks before and after harmonization obtained by GMaps and OSM tools for the 16 cities with official spatial data.

Source	Region	State^1^	Capital	Official source		Conservation Units		Harmonized official data				GMaps and OSM					OSM				
				N	Area (km^2^)	N	Area (km^2^)	N	(%)	Area (km^2^)	(%)	N	(%)	Area (km^2^)	(%)	Agreement^4^ %	N	(%)	Area (km^2^)	(%)	Agreement^4^%
**Official**	Mid-West	DF	Brasília	56	81.5	43	52.2	13	(4.3)	29.8	(20.5)	0	(0.0)	0.0	(0.0)	0.0	3	(1.3)	1.5	(3.7)	23.1
	North	AP	Macapá	1	0.7	-	-	1	(0.3)	0.7	(0.5)	1	(0.4)	0.6	(1.9)	100.0	0	(0.0)	0.0	(0.0)	0.0
		RO	Porto Velho	2	0.1	-	-	2	(0.7)	0.1	(0.1)	2	(0.7)	0.1	(0.3)	100.0	2	(0.9)	0.1	(0.2)	100.0
		TO	Palmas^2^	8	17.1	-	-	2	(0.7)	2.1	(1.4)	2	(0.7)	0.4	(1.3)	100.0	2	(0.9)	0.4	(1.0)	100.0
	Northeast	AL	Maceió	1	1.8	-	-	1	(0.3)	1.8	(1.2)	1	(0.4)	1.2	(3.8)	100.0	0	(0.0)	0.0	(0.0)	0.0
		BA	Salvador	29	57.0	19	33.1	10	(3.3)	16.9	(11.6)	1	(0.4)	0.6	(1.9)	10.0	3	(1.3)	0.7	(1.7)	30.0
		CE	Fortaleza	27	10.8	2	5.0	25	(8.3)	5.8	(4.0)	9	(3.3)	0.3	(1.0)	36.0	20	(8.7)	4.9	(11.8)	80.0
		MA	São Luís	5	32.7	3	36.4	2	(0.7)	2.0	(1.4)	1	(0.4)	0.0	(0.0)	50.0	2	(0.9)	0.1	(0.2)	100.0
		PE	Recife	19	1.0	4	14.6	15	(5.0)	1.0	(0.7)	6	(2.2)	0.3	(1.0)	40.0	14	(6.1)	0.7	(1.7)	93.3
		RN	Natal	2	12.9	1	1.6	1	(0.3)	11.4	(7.9)	1	(0.4)	10.8	(34.4)	100.0	1	(0.4)	1.4	(3.4)	100.0
		SE	Aracaju	5	4.5	1	1.8	4	(1.3)	2.8	(1.9)	3	(1.1)	2.0	(6.4)	75.0	3	(1.3)	2.0	(4.9)	75.0
	South	PR	Curitiba^3^	74	14.6	1	0.1	47	(15.6)	14.3	(9.9)	28	(10.4)	3.6	(11.5)	59.6	43	(18.7)	7.2	(17.7)	91.5
		RS	Porto Alegre	9	2.4	-	-	9	(3.0)	2.4	(1.6)	8	(3.0)	2.2	(7.0)	88.9	9	(3.9)	2.4	(5.9)	100.0
	Southeast	ES	Vitória^3^	70	52.5	19	49.5	11	(3.6)	0.6	(0.4)	7	(2.6)	0.4	(1.3)	63.6	8	(3.5)	0.5	(1.2)	72.7
		MG	Belo Horizonte^3^	87	9.5	-	-	75	(24.8)	8.8	(6.1)	39	(14.4)	3.4	(10.8)	52.0	42	(18.3)	3.6	(8.8)	56.0
		SP	São Paulo	98	106.7	13	112.1	84	(27.8)	22.1	(15.2)	33	(12.2)	5.5	(17.5)	39.3	78	(33.9)	15.3	(37.6)	92.9
			Total	493	405.8	106	306.3	302	100.0	122.6	100.0	142	100.0	31.4	100.0	47.0	230	100.0	40.8	100.0	76.2

1 AL: Alagoas; AP: Amapá; BA: Bahia; CE: Ceará; DF: Distrito Federal; ES: Espírito Santo; MA: Maranhão; MG: Minas Gerais; PE: Pernambuco; PR: Paraná; RS: Rio Grande do Sul; RN: Rio Grande do Norte; RO: Rondônia; SE: Sergipe; SP: São Paulo; TO: Tocantins.

2 Data contained planned parks, only two parks are implemented in the city.

3 Cities where other types of green areas were extracted in addition to Conservation Units

4 Concordance percentage, calculated as the number of concordance parks divided by the total number of harmonized parks.

Table 2 presents the descriptive analysis of the 11 cities with non-official spatial data. For five of the eleven cities, textual information (such as address, name, coordinates, and description) was combined with GMaps-OSM to find the parks. For the other six cities we relied only on GMaps-OSM results. For this set of eleven cities, we found 128 parks with a total area of 22.5 km^2^.

**Table 2:** Number and area of urban parks obtained by GMaps and OSM tools for the 11 cities with non-official sources.

Source	Region	State^1^	Capital	N	(%)	Area (km^2^)	(%)
**Textual information + GMaps-OSM^2^**	Mid-West	GO	Goiânia	47	(17.4)	5.29	(23.5)
	North	AC	Rio Branco	11	(4.1)	3.5	(15.6)
	Northeast	PI	Teresina	6	(2.2)	0.7	(3.1)
	South	SC	Florianópolis	5	(1.9)	0.5	(2.1)
	Southeast	RJ	Rio de Janeiro	21	(7.8)	5.3	(23.7)
			Subtotal	90	(70.3)	15.3	(68.0)
**Gmaps-OSM**	Mid-West	MS	Campo Grande	9	(3.3)	2.6	(11.3)
		MT	Cuiabá	5	(1.9)	0.7	(3.2)
	North	AM	Manaus	13	(4.8)	1.5	(6.5)
		PA	Belém	3	(1.1)	0.3	(1.1)
		RR	Boa Vista	3	(1.1)	1.3	(5.8)
	Northeast	PB	João Pessoa	5	(1.9)	0.9	(4.0)
			Subtotal	38	(29.7)	7.2	(32.0)
			Total	128	(100.0)	22.5	(100.0)

^1^ AC: Acre; AM: Amazonas; GO: Goiás; MS: Mato Grosso do Sul; MT: Mato Grosso; PA: Pará; PB: Paraíba; PI: Piauí; RJ: Rio de Janeiro; RR: Roraima; SC: Santa Catarina. ^2^ City where other types of green areas were extracted in addition to Conservation Units; ^2^ Searches on GMaps and OSM were supported by textual information provided by city halls.

After verification and harmonization, the complete dataset (official and GMaps-OSM) included 430 urban parks with a total area of 145.14 km^2^. The mean number of parks across cities was 16, with a mean size area of 0.33 km^2^. The median number of parks was nine, with a median area of 0.07 km^2^, demonstrating that most capitals sampled have less than six parks (S2 Fig).

### 3.2 Comparison between data sources

Of the 302 urban parks identified from the harmonized official data, 42 (47%) were located using GMaps-OSM, and 230 (76.2%) using only OSM (Table 1). The OSM presented a better percentage of agreement with the harmonized official data; however, the agreement varied between the capitals. No parks were found in Maceió and Macapá; both capitals had only one official park. For five capitals, OSM detected 100% of the parks (Porto Velho, Palmas, São Luís, Natal and, Porto Alegre). The agreement varied from 72.7% (Vitória) to 93.3% (Recife), for eight capitals. Only three capitals had agreement below 60.0%, namely Brasília (23.1%), Salvador (30%), and Belo Horizonte (56%).

Table 3 presents the concordance analysis between park areas obtained by harmonized official data and OSM, totaling 230 parks stratified by capital. Maceió, Macapá and Natal cities were not included in the analysis because they had only one park.

**Table 3:** Reliability of urban park areas between official sources and OSM for the 16 cities with official sources.

Source	Region	State^1^	Capital	N	Area (km^2^)		ICC (CI95%)
					Harmonized official data	OSM	
**Official**	Mid-West	DF	Brasília	3	1.4	1.5	0.71 (0.00; 0.99)
	North	AP	Macapá	1	0.7	-	-
		RO	Porto Velho	2	0.1	0.1	0.99 (0.94; 0.99)*
		TO	Palmas	2	2.1	0.4	0.00 (0.00; 0.99)
	Northeast	AL	Maceió	1	1.8	-	-
		BA	Salvador	3	4.0	0.7	0.00 (0.00; 0.90)
		CE	Fortaleza	20	4.5	4.8	0.98 (0.96; 0.99)*
		MA	São Luís	2	2.1	0.1	0.00 (0.00; 0.99)
		PE	Recife	14	0.9	0.7	0.60 (0.14; 0.85)*
		RN	Natal	1	11.4	1.4	-
		SE	Aracaju	3	2.7	2.0	0.92 (0.26; 0.99)*
	South	PR	Curitiba	43	13.0	7.2	0.53 (0.28; 0.71)*
			Curitiba^2^	42	6.8	5.5	0.84 (0.73; 0.91)*
		RS	Porto Alegre	9	2.4	2.4	0.97 (0.89; 0.99)*
	Southeast	ES	Vitória	8	0.4	0.5	0.67 (0.06; 0.92)*
		MG	Belo Horizonte	42	6.5	3.6	0.13 (0.00; 0.42)
			Belo Horizonte^2^	40	3.3	3.5	0.92 (0.86; 0.95)*
		SP	São Paulo	78	19.3	15.3	0.25 (0.03; 0.45)*
			São Paulo^2^	74	11.6	9.4	0.98 (0.97; 0.99)*

ICC–Intraclass correlation; CI95%—Confidence Interval 95%; *p<0.05; ^1^ AL: Alagoas; AP: Amapá; BA: Bahia; CE: Ceará; DF: Distrito Federal; ES: Espírito Santo; MA: Maranhão; MG: Minas Gerais; PE: Pernambuco; PR: Paraná; RS: Rio Grande do Sul; RN: Rio Grande do Norte; RO: Rondônia; SE: Sergipe; SP: São Paulo; TO: Tocantins. ^2^ Without outliers: Curitiba (1), Belo Horizonte (2), São Paulo (4)

The agreement was considered excellent (ICC greater than 0.90) for the cities of Porto Velho, Fortaleza, Aracajú, and Porto Alegre, moderate (ICC between 0.5 and 0.75) in Recife, Curitiba, and Vitória and poor in Palmas, Salvador and São Luís (ICC = 0), Belo Horizonte (ICC = 0.13) and São Paulo (ICC = 0.25). The ICC was not significant for Brasília, Palmas, Salvador, São Luís, and Belo Horizonte, indicating the lack of agreement between the sources. For Curitiba, Belo Horizonte, and São Paulo, the analysis was redone excluding, respectively, one, two and four parks whose official areas were much larger than the areas obtained by OSM, which were identified from scatterplots (S3 Fig). After the exclusions, the agreement was excellent for all.

For the capitals not showing excellent agreement (Palmas, Salvador, São Luís, Recife, Curitiba, Belo Horizonte, and São Paulo), the area obtained by the harmonized official source was greater than the ones obtained by OSM, with an exception of Brasilia and Vitória (Table 3). Discrepancies in results between OSM and official data may be related to the area available for visitation or experienced by users. We found that some of these parks had large green areas limiting the visitation area of the users and report of park area (S4 Fig).

## 4. Discussion

Research addressing the importance of urban parks for health is essential for planning more sustainable cities. It is also very relevant for regions such as Latin America, with high urbanization rates. Therefore, information about parks’ presence, location, and area is highly relevant. Our findings point to cross-city differences in the spatial distribution of urban parks in Brazil, official data availability and non-official sources used to collect the data. We found greater accuracy between official harmonized spatial data and OSM data sources. The lack of data access impacts research on urban parks. To this end, the present study exemplified the application of a methodology to access, collect and harmonize spatial data from urban parks in Brazilian capitals proposing tools to bridge this gap. The use of these tools can help to increase the availability and improve the quality of this information.

Challenges linked to the lack of accuracy and information availability are present in official or collaborative data. The first steps in obtaining spatial data from urban parks were accomplished by consulting publicly accessible sources for each Brazilian capital and direct contact with city halls without online data. In our study, we obtained spatial data for 16 cities, and we received textual data for five cities. For the remaining six cities, we did not receive any official data. We cannot confirm that there is no official data, but only that the data was not accessible.

Federal law mandates Brazilian government entities to deliver the information requested by citizens. According to Michener et al. [28], Brazilian municipal entities are less likely to meet these requests than the States and Federal governments. Their study found an official response rate of 68% and an accuracy rate of 59.6% for 11 Brazilian capitals. In our research, 21 requests were made through e-SIC, but 11 (or 52.4%) did not receive any response. Compliance with the Access to Information Law in Brazil is still a challenge and can hinder scientific research and public data collection. Failure to receive the requested data suggested an inexistence of data, maybe due to a lack a local cartographic sector.

The comparison analysis showed that the combination of GMaps-OSM did not provide a comprehensive list of parks compared to data from local government authorities. It can be partly because the GMaps platform has a restriction of 120 results per search, which can limit the number of parks returned in the search results. Other elements, such as commercial establishments, parking lots, and condominiums, were also included in the results. This may be attributed to the high relevance of these elements in the GMaps search results and a possible classification problem of these same elements on this platform. As a result of these issues, the number of parks could have been hidden, and therefore their number reduced. In addition, the lack of filter options in the GMaps search platform could also have contributed to the reduced number of urban parks.

To obtain a more accurate spatial dataset of urban parks, the local government entity must provide at least an official textual list. Comparison of harmonized official data with OSM data showed a higher degree of agreement than the GMaps-OSM method, showing concordance of 76.2% and 42%, respectively. It indicates that even when no official spatial data is available, having official textual data along with OSM is important for a more precise representation of urban parks. While the GMaps method provides only the location in geographical coordinates (points), OSM provides park delimitation in polygons. This is essential for studies that require information about the extent and characterization of parks.

The comparison analysis showed discrepancies in the delimitation and size of park areas between OSM and official data. This may be due to the limited space available for visitation or experience by users who map what is perceived. As shown in S4 Fig, most of these parks comprise large green areas and a small built area (playground, parking lot, bathroom, sport court, track, and others). In these cases, the OSM polygon delimits only the built space. In general, OSM park areas were more minor than official data. This difference was even more remarkable in cities where there was no agreement between the sources in the ICC evaluation or when the agreement was poor.

OSM and GMaps provide an important and affordable tool to fill up urban park data gaps and are found to capture urban landscape changes and accessibility adequately [29, 30]. Nonetheless, one should consider the collaborative nature of the data from OSM. Depending on the location, the accuracy and completeness of data can vary, demanding a certain level of precaution since, in some cases, the park polygons may not have a good positioning or cannot be found if we compared them with a reference dataset [31–33]. However, as done in this study, the development of methodological approaches to verify the quality of these platforms is essential to support and improve data provision when official data is unavailable. The limitations of the GMaps and OSM methods identified in this study suggest that combining official data with OSM data may be more effective in obtaining a more comprehensive and accurate representation of urban parks.

Another challenge appears to be the lack of a standard interdisciplinary definition that captures what constitutes an urban park and how concepts, purposes, and usage of urban parks have evolved over the years from aesthetics to the environmental health realm [3, 6, 34].

## 5. Conclusion

This study highlights the importance of creating mechanisms to access, collect, verify, and harmonize urban park data that can contribute to better and more equitable provisions of urban parks and inform studies on urban health. It also points out the need to differentiate and define what constitutes an urban park and its differences from other greenspaces often mistaken for urban parks in data collection. The proposed procedures constitute the first step to foster studies related to urban parks, particularly at the intra-urban level, and show the importance of public open access data for policymakers, civil society, and researchers. Urban parks greatly benefit health, particularly for physical activity, and help mitigate climate change impacts in cities. Encouraging such studies would contribute to policymakers developing initiatives integrating parks’ development into urban sustainability.

## Supporting information

S1 Table Geographic characteristics of Brazilian capitals. (DOCX)

S2 Table Number of CSUs by capitals and regions. Official source in Brazil (SNUC).
(DOCX)

S1 Fig Urban parks and conservation units for the municipalities of Brasília, Salvador and Rio de Janeiro. (DOCX)

S2 Fig Descriptive statistics of the number and area (km2) of urban parks in Brazilian capitals. (DOCX)

S3 Fig Scatterplots of urban park areas (km^2^) between official sources and OSM for the 14 cities with OSM source. (DOCX)

S4 Fig Delimitation of urban parks from official and OSM data. (DOCX)

S5 Fig Map with the number of parks in Brazilian capitals. (DOCX)
