# Supplementary material for: Spatial data collection and qualification methods for urban parks in Brazilian capitals: An innovative roadmap
Source: PLoS One. 2023 Aug 10;18(8):e0288515. doi: 10.1371/journal.pone.0288515 (PMC10414613; doi:10.1371/journal.pone.0288515)
Supplement: S1 Table — (DOCX) [file pone.0288515.s001.docx]

**S1 Table. Geographic Characteristics of Brazilian capitals**

| **Region** | **State** | **Capital** | **Climate Zone** * | **Main Biome / Altimetry (meter) **** | **City area (km²)** |
| --- | --- | --- | --- | --- | --- |
| **North** | AC | Rio Branco | Equatorial | Tropical Rainforest / 110 - 348 m | 10,430.30 |
|  | AP | Macapá | Equatorial | Tropical Rainforest / 21 - 110 m | 6,468.00 |
|  | AM | Manaus | Equatorial | Tropical Rainforest / 21 - 176 m | 12,244.30 |
|  | PA | Belém | Equatorial | Tropical Rainforest / 0 - 21 m | 1,062.50 |
|  | RO | Porto Velho | Equatorial | Tropical Rainforest / 21 - 409 m | 38,175.50 |
|  | RR | Boa Vista | Equatorial | Tropical Rainforest / 21 a 409 m | 6,134.20 |
|  | TO | Palmas | Central Brazil Tropical | Savanna / 176 - 732 m | 2,223.70 |
| **Northeast** | AL | Maceió | Eastern Northeast Tropical | Tropical Rainforest / 0 - 300 m | 515.80 |
|  | BA | Salvador | Eastern Northeast Tropical | Tropical Rainforest / 0 - 110 m | 701.30 |
|  | CE | Fortaleza | Equatorial Zone Tropical | Scrubland / 0 - 57 m | 318.70 |
|  | MA | São Luís | Equatorial Zone Tropical | Tropical Rainforest / 0 - 84 m | 834.20 |
|  | PB | João Pessoa | Eastern Northeast Tropical | Tropical Rainforest / 0 - 84 m | 218.00 |
|  | PE | Recife | Eastern Northeast Tropical | Tropical Rainforest / 0 - 110 m | 225.00 |
|  | PI | Teresina | Equatorial Zone Tropical | Savanna / 21 - 255 m | 1,393.00 |
|  | RN | Natal | Eastern Northeast Tropical | Scrub and Savanna / 0 - 110 m | 172.10 |
|  | SE | Aracaju | Eastern Northeast Tropical | Tropical Rainforest / 0 - 110 m | 185.10 |
| **Midwest** | GO | Goiânia | Central Brazil Tropical | Savanna / 700 - 890 m | 736.00 |
|  | MT | Cuiabá | Central Brazil Tropical | Savanna / 141 - 850 m | 3,614.30 |
|  | MS | Campo Grande | Central Brazil Tropical | Savanna / 213 - 485 m | 8,274.40 |
|  | DF | Brasília | Central Brazil Tropical | Savanna / 750 - 1100 m | 5,788.30 |
| **Southeast** | ES | Vitória | Central Brazil Tropical | Tropical Rainforest / 0 - 300 m | 99.30 |
|  | MG | Belo Horizonte | Central Brazil Tropical | Savanna and Tropical Rainforest / 650 - 1000 m | 331.20 |
|  | RJ | Rio de Janeiro | Central Brazil Tropical | Tropical Rainforest / 5 - 980m | 1,200.10 |
|  | SP | São Paulo | Central Brazil Tropical | Tropical Rainforest / 80 - 1000 m | 1,520.90 |
| **South** | PR | Curitiba | Temperate | Tropical Rainforest / 700 - 930 m | 436.70 |
|  | RS | Porto Alegre | Temperate | Grassland / 5 - 300 m | 676.90 |
|  | SC | Florianópolis | Temperate | Tropical Rainforest / 0 - 480 m | 500.60 |

^*^ Climate map of Brazil, Rio de Janeiro: IBGE, 2002. 1 map. Scale 1:5 000 000.

Available at: http://geoftp.ibge.gov.br/informacoes_ambientais/climatologia/mapas/brasil/Map_BR_clima_2002.pdf. Accessed on out. 2021.

^**^ According to IBGE vegetation classification.
