# Supplementary material for: Spatial data collection and qualification methods for urban parks in Brazilian capitals: An innovative roadmap
Source: PLoS One. 2023 Aug 10;18(8):e0288515. doi: 10.1371/journal.pone.0288515 (PMC10414613; doi:10.1371/journal.pone.0288515)
Supplement: S2 Table — Official source in Brazil (SNUC). (DOCX) [file pone.0288515.s002.docx]

**S 2 Table. Number of CSUs by capitals and regions. Official source in Brazil (SNUC).**

| **Region** | **Capital** | **Conservation units** | **Area (Km²)** |
| --- | --- | --- | --- |
| North | Rio Branco | 3 | 9727.4 |
|  | Macapá | 1 | 221.7 |
|  | Manaus | 7 | 11812.3 |
|  | Belém | 3 | 86.0 |
|  | Porto Velho | 10 | 10496.7 |
|  | Boa Vista | 1 | 0.5 |
|  | Palmas | 2 | 1234.1 |
| **Total** | | **26** | 33578.7 |
| Northeast | Maceió | 3 | 137.2 |
|  | Salvador | 2 | 24.3 |
|  | Fortaleza | 6 | 56.2 |
|  | São Luís | 4 | 58.7 |
|  | João Pessoa | 4 | 12.6 |
|  | Recife | 3 | 346.1 |
|  | Teresina | 0 | 0.0 |
|  | Natal | 1 | 19.0 |
|  | Aracaju | 1 | 1.7 |
| **Total** | | **24** | 655.8 |
| Midwest | Goiânia | 1 | 749.0 |
|  | Cuiabá | 5 | 3638.0 |
|  | Campo Grande | 2 | 3.1 |
|  | Brasília | 30 | 7984.4 |
| **Total** | | **38** | 12374.6 |
| Southeast | Vitória | 12 | 25.1 |
|  | Belo Horizonte | 3 | 1697.7 |
|  | São Paulo | 16 | 456.7 |
|  | Rio de Janeiro | 50 | 476.1 |
| **Total** | | **81** | 2655.6 |
| South | Curitiba | 3 | 199.4 |
|  | Porto Alegre | 0 | 0.2 |
|  | Florianópolis | 10 | 104.3 |
| **Total** | | **13** | 303.9 |
| **Total Final** | | **182** | 49568.5 |
