# Supplementary figures and images for: Spatial data collection and qualification methods for urban parks in Brazilian capitals: An innovative roadmap
Source: PLoS One. 2023 Aug 10;18(8):e0288515. doi: 10.1371/journal.pone.0288515 (PMC10414613; doi:10.1371/journal.pone.0288515)

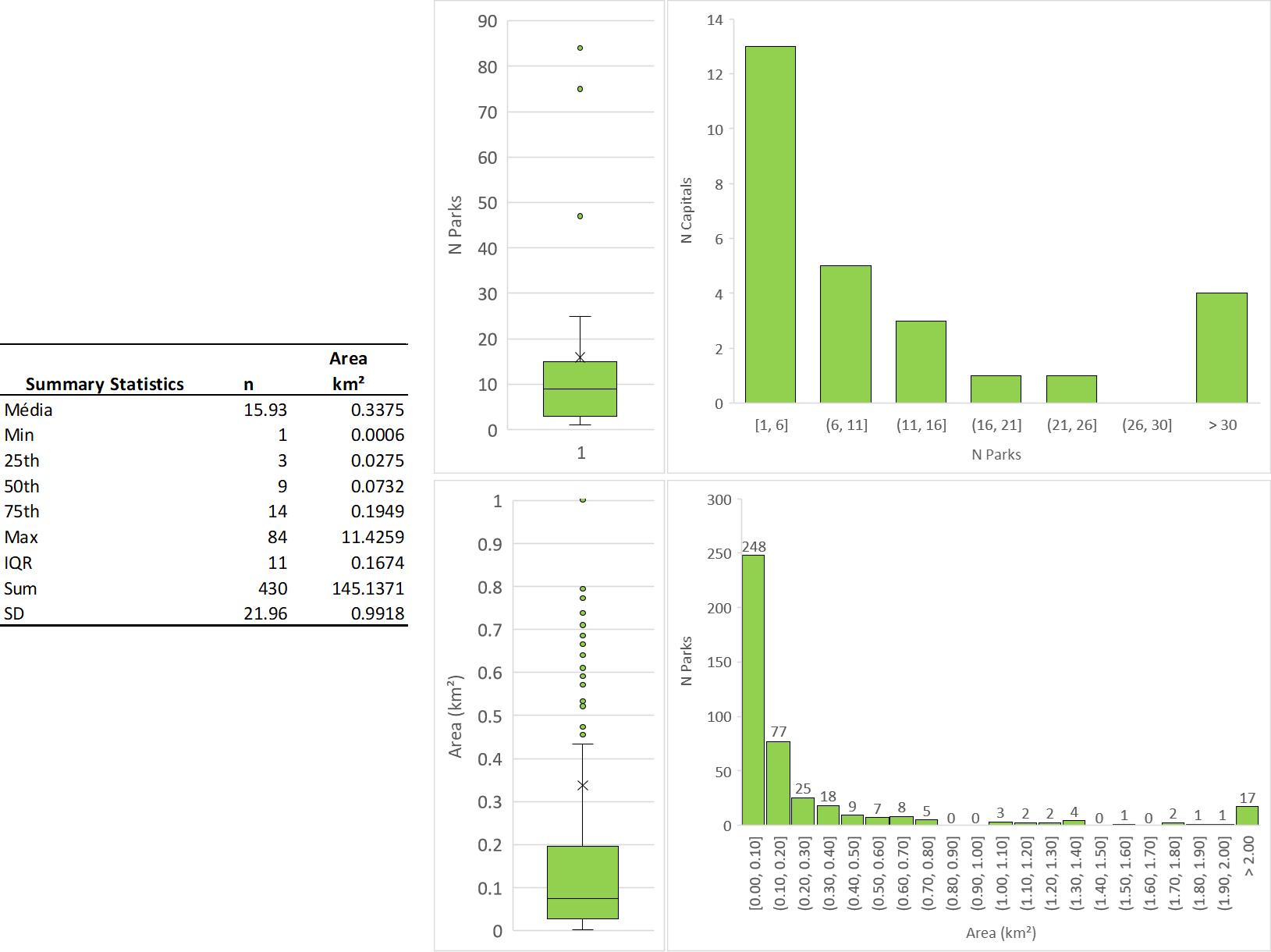


**S2 Figure. Descriptive statistics of the number and area (km2) of urban parks in Brazilian capitals**

Supplement: S2 Fig — (DOCX) [file pone.0288515.s004.docx]

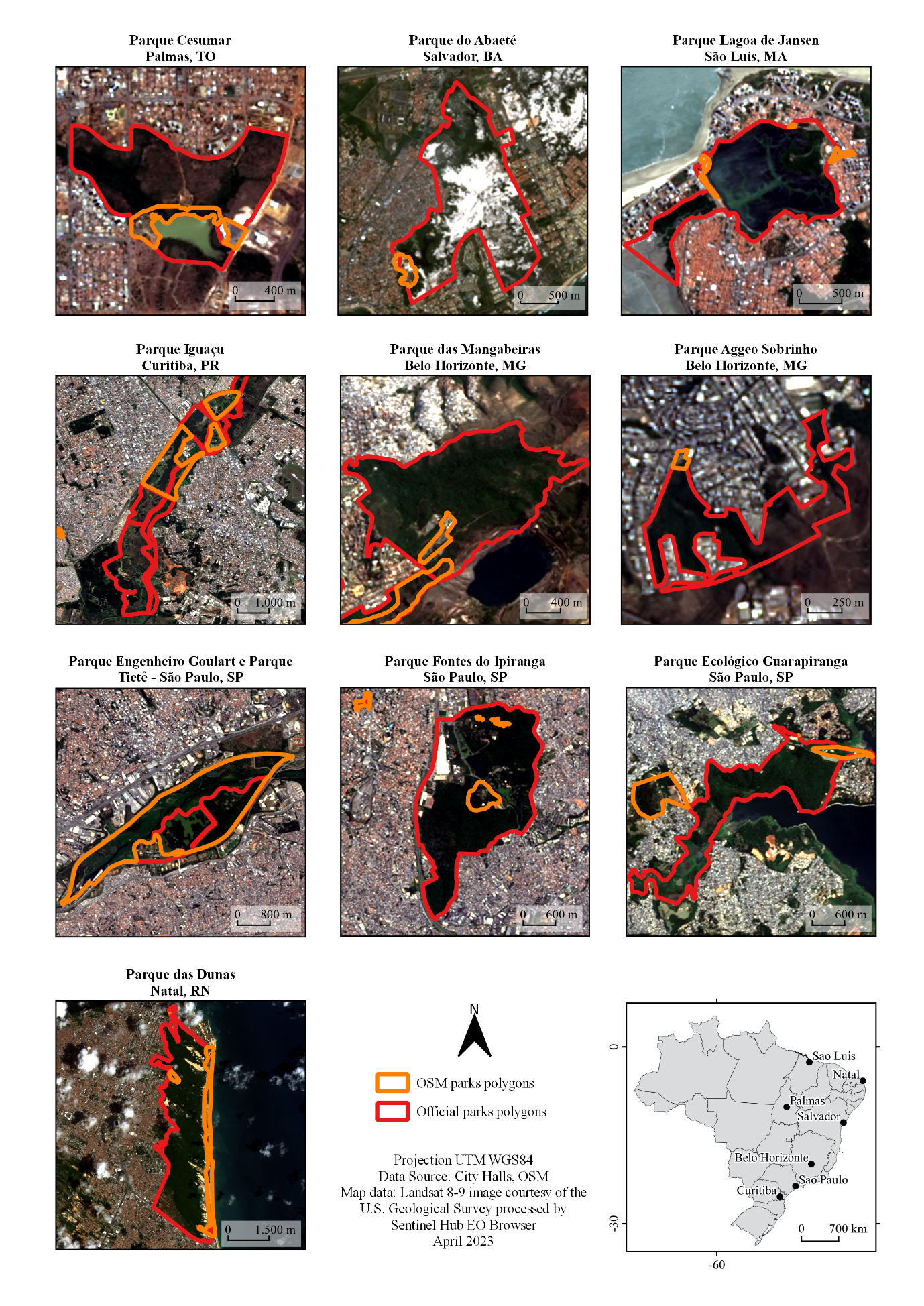


**S4 Figure. Delimitation of urban parks from official and OSM data**

Supplement: S4 Fig — (DOCX) [file pone.0288515.s006.docx]

**
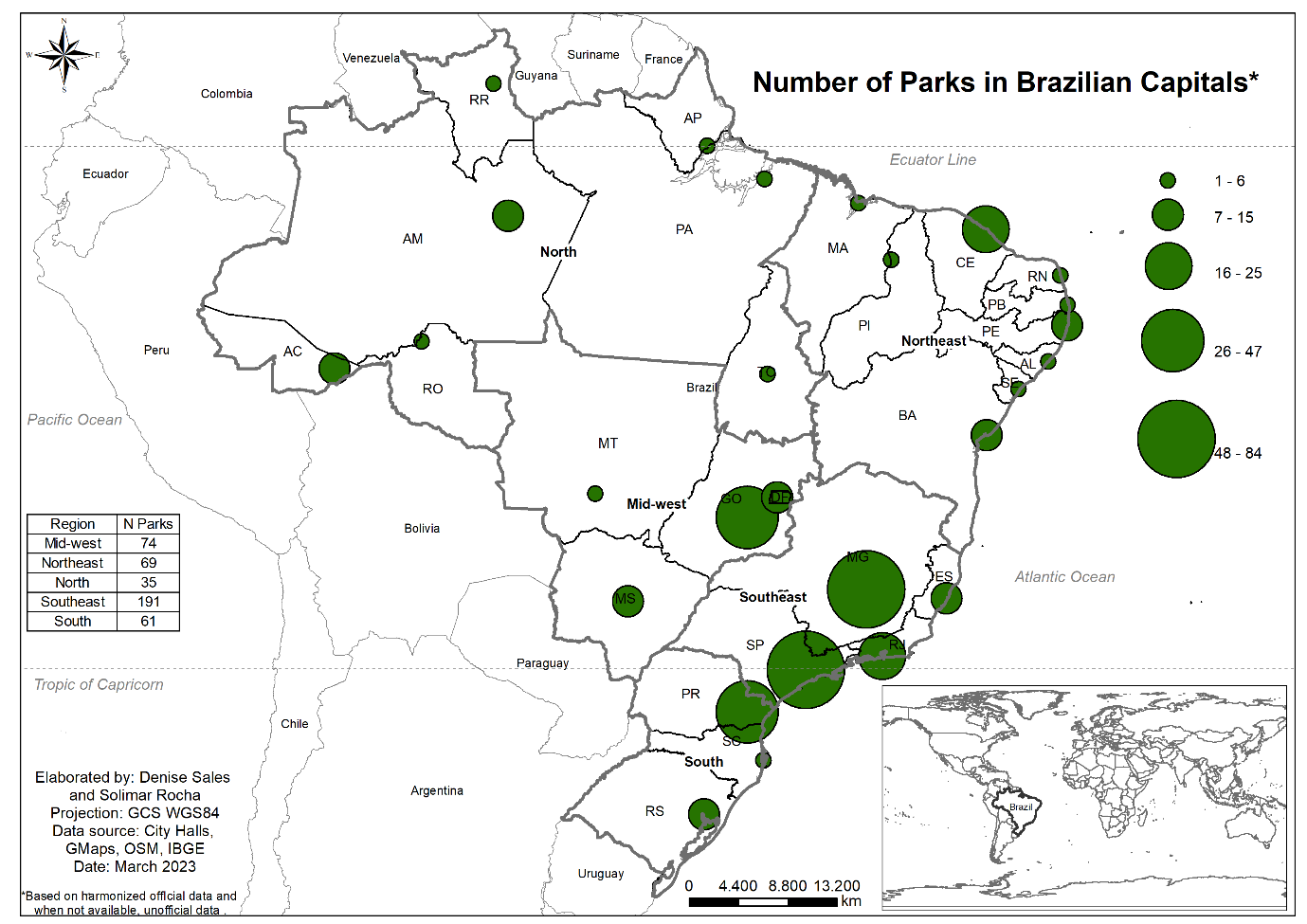
**

**S5 Figure. Map with the number of parks in Brazilian capitals**

Supplement: S5 Fig — (DOCX) [file pone.0288515.s007.docx]
