# Supplementary material for: Spatial data collection and qualification methods for urban parks in Brazilian capitals: An innovative roadmap
Source: PLoS One. 2023 Aug 10;18(8):e0288515. doi: 10.1371/journal.pone.0288515 (PMC10414613; doi:10.1371/journal.pone.0288515)
Supplement: S3 Fig — (DOCX) [file pone.0288515.s005.docx]

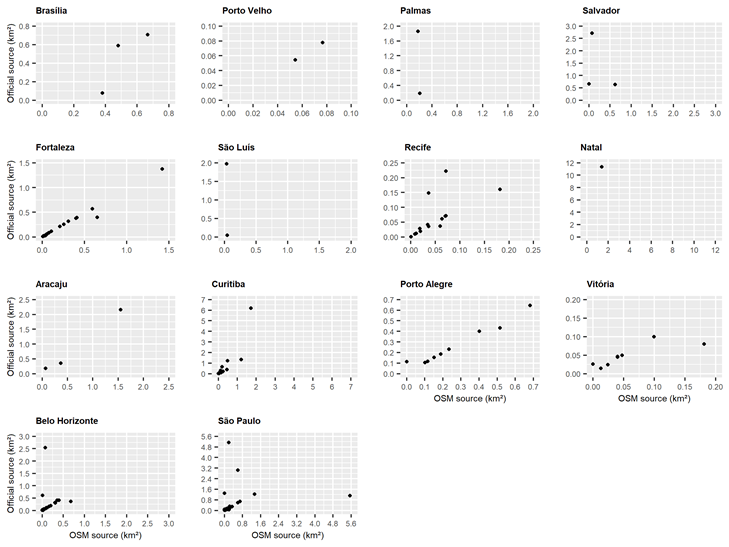


**S3 Figure. Scatterplots of urban park areas (km²) between official sources and OSM for the 14 cities with OSM source**
